# Unstable employment and health in middle age in the longitudinal 1970 British Birth Cohort Study

**DOI:** 10.1093/emph/eoy009

**Published:** 2018-03-27

**Authors:** David Waynforth

**Affiliations:** School of Medicine, Faculty of Health Sciences and Medicine, Bond University, Gold Coast, QLD 4229, Australia

**Keywords:** stochastic, gig economy, chronic conditions, life history strategies, obesity

## Abstract

**Background and objectives:**

Jobs for life have become increasingly rare in industrialized economies, and have been replaced by shorter-term employment contracts and freelancing. This labour market change is likely to be accompanied by physiological changes in individuals who have experienced little job stability. Evolved responses to increased environmental instability or stochasticity include increased fat deposition and fight-or-flight responses, such as glucose mobilization and increased blood pressure. These responses may have evolved by natural selection as beneficial to individuals in the short-term, but are damaging in the longer term.

**Methodology:**

This study tested whether job losses experienced between ages 30 and 42 are associated with increased body weight, hypertension and diabetes diagnosis in the 1970 British Birth Cohort, which consists of all registered births in a one-week period in April 1970.

**Results:**

Each job loss experienced increased the odds of developing diabetes by 1.39 times (CI 1.08–1.80), and of hypertension by 1.28 times (CI 1.07–1.53). Another economic variable, higher personal debt, was associated with all three of these health outcomes: every £100 000 of debt roughly doubled the odds of gaining at least 5 kg between ages 30 and 42.

**Conclusions and implications:**

These associations between job loss and health-risk factors suggest that our changing economy results in increases in the prevalence of risk factors for cardiovascular disease. At a broader level, they are consistent with evolutionary understandings of environmental stochasticity, and are a reminder that economic policy is also health policy.

## Teaser

Evolutionary theory predicts increased fat storage and stress in response to unstable environments. Consistent with this, in a sample of middle-aged men and women in the UK, number of job losses experienced between ages 30 and 42 was associated with increased odds of diabetes diagnosis and high blood pressure. Evidence for an association between job losses and weight gain was less conclusive.

## BACKGROUND AND OBJECTIVES

Generation X, those of us born in the 1960s and 1970s, have entered middle age in globalized economic environments in which jobs for life are no longer as commonplace as they were for the previous generation [1, 2]. Many careers characterized by long term employment in the past have been replaced by freelancing, casual labour, zero-hour and short term contracts often referred to as the ‘gig’ economy [3].

The gig economy results in fluctuations or stochasticity in resource availability for individuals. Experiencing stochasticity is not novel for humans or other animals, and has been studied from an evolutionary perspective using mathematical models, experimental and observational studies. Optimality modelling has indicated that it is typically evolutionarily optimal to allocate more energy to storage (including fat) as an environment becomes more stochastic [4]. Consistent with this, observed plasticity in energy storage as a function of changing resource availability has been demonstrated in a number of species [5–7].

A metabolic shift towards fat storage in increasingly stochastic environments may provide an adaptive buffer against future food shortage, but comes at a cost to long term health when it results in obesity [8]. Other effects of stochasticity are also less likely to be beneficial in the long term: financial and food instability lead to increased psychological stress including elevated cortisol levels [9]. Elevated cortisol as a response to a crisis or threat situation is likely to have evolved as part of an adaptive suite of neuroendocrine responses which motivate an individual to take appropriate actions to cope with the threat [10, 11]. In the longer term, chronic or repeated exposure to cortisol is associated with hypertension and developing Type 2 diabetes, among other damaging effects [12–14].

In humans, two lines of evidence that stochastic environments result in increased stress and body weight are from studies of poverty and obesity, and of perceived employment instability and health-related variables. Financial insecurity or poverty severe enough to affect the ability to reliably purchase food in industrialized economies is frequently observed to be associated both with obesity and behaviours which lead to obesity [15–18]. Studies of impacts of job insecurity on health have shown evidence of associations between perceived job insecurity and various health outcomes, including mental health, stress and cardiovascular risk factors [19–22]. To date most studies of job insecurity and health outcomes have relied on threatened and self-reported perceived job insecurity rather than actual job losses experienced ([19–22] and references therein). The problem with this approach is that there may be many individuals who have an unwarranted belief their job security is low, as well as individuals not prone to worrying about job security when their job is actually at risk. Hence, perceived job insecurity is also measuring an element of neuroticism which itself may be associated with health outcomes, and is not a measure of an individual’s experience of stochasticity [23].

The present study addresses the question of how job instability may have impacted health in early middle age in the 1970 longitudinal British Birth Cohort, for which detailed data are available on employment histories and on personal finances, as well as several health-related variables relevant to the hypothesis that job insecurity will lead to adverse health outcomes. Body weight data were collected in several data sweeps, and the most recent interview (2012) contained questions on whether the cohort members had developed high blood pressure and diabetes in middle-adulthood.

## METHODOLOGY

The sample was drawn from the British longitudinal cohort study of all children born in a one-week period in April 1970 (17 287 babies). The last follow-up for these individuals occurred in 2012, when the cohort study participants were 42 years old. For inclusion in the study sample for the present research, participants must have completed the employment survey and have provided health status information in the interview. Around half of the original sample have been lost to follow-up or did not complete these surveys. Generally, most loss to follow-up in this cohort has been due to failure to trace individuals who have moved address [24]. Further information on the sample, survey design, and the format of interviews and questions are available from the Centre for Longitudinal Studies [25], and a cohort profile has also been published [24].

### Variables

Three dependent variables were selected to test the hypothesis: a reported diagnosis of high blood pressure, diabetes, and of a change in body weight between ages 30 and 42. The main independent variable of interest was number of job losses experienced by the cohort member between 2000 and 2012. Categories of reasons for job loss included in the job losses variable are shown in Table 1. Categories of job loss were selected which best represent stochasticity: short-term contracts ending, work hours being cut, businesses failing and roles being eliminated resulting in redundancy. Because some employment consists of working for a business as an independent contractor rather than directly as an employee, leaving employment due to lack of work was included. The job loss variable excludes being dismissed or fired from jobs, as health problems could lead to dismissal, thus potentially confusing cause and effect in the study.

**Table 1.** Reasons given by cohort members for redundancies/job losses included in the job losses variable

Main reason employment ended	Count
Fixed term or temporary job ended	351
Made redundant	880
Firm closed down/business failed	233
Lack of job security/lack of work	60
Reduced working hours	77
Business taken over by another company	44
Total	1645

Health-related effects of job loss are likely to be less evident if an individual has savings to fall back on after losing a job, and has little debt on which payments must be made. For this reason, reported savings and debts were included as covariates in the regression models. The 2012 sweep included questions about finances, but the 2000 sweep did not, hence savings and debt at the end of the study period were included as variables. Mortgages were not included in the debt variable, but all other debt was included, such as car hire purchase and credit card debt. Home value was not included in the savings variable. Savings and debt are highly correlated with the income-profession-based measures of socioeconomic status used the 1970 birth cohort, thus socioeconomic status derived from employment was not included as a covariate. Instead, whether or not the cohort member has a university degree was included as a binary control for socio-economic status.

Four measures of lifestyle from the 2012 sweep were included as control variables: reported number of cigarettes smoked per day; reported frequency of alcohol consumption per week, using a scale from 1 to 5 where 5 is high (four times per week or more); number of times per week the participant reported doing 30 min or more of exercise; and number of times per week the participant reported eating fruit and vegetables (cooked and uncooked). Although no prediction was made regarding different effects of job loss on men and women, sex was included as a covariate.

### Statistical analysis

**Figure 1.**
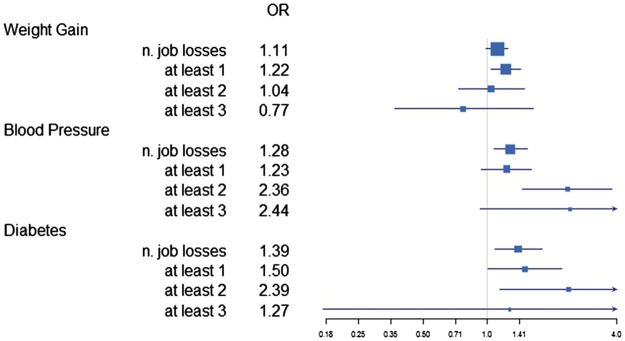
Forest plot showing the main study results and results of sensitivity analyses exploring whether number of job losses or a threshold number of job losses best explains the relationship between job loss and health risk factors. The binary job loss variables were entered into logistic regression models including all covariates shown in Tables 4–6

**Figure 2.**
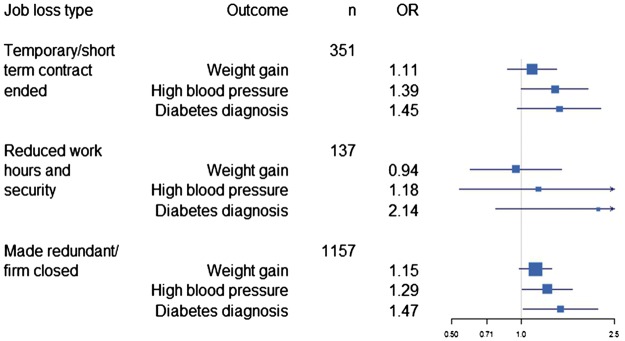
Forest plot showing results of analyses exploring whether the different categories of job loss included in the job loss variable have different effects on health outcomes. The first category shown represents the first category in Table 1; the second category is the fourth and fifth in Table 1 and the third consists of the remaining categories shown in Table 1. The binary job loss variables were entered into logistic regression models including all covariates shown in Tables 4–6

Multiple logistic regression was used for all three inferential statistical tests, using SPSS Statistics Version 24. Figs 1 and 2 were produced using the Forestplot package in RStudio 1.0153. Weight gain was transformed into a binary variable for analysis (see below). Two additional sets of analyses were carried out following the main logistic regression models: sensitivity analyses were carried out to determine whether there is a threshold at which number of job losses has an association with the outcome: for example, it is possible that a single job loss has little or no effect on health outcomes, but that only multiple job losses affect health outcomes. Second, the job loss categories were further broken down for analysis of whether fixed-term contracts ending have different effects on the health measures compared with being made redundant before the end of an employment contract.

## RESULTS

### Descriptive statistics

Of 9841 cohort members who participated in 2000 and 2012, 5904 participants had provided sufficiently complete information across all variables for inclusion in regression analyses of blood pressure and diabetes. The weight gain variable had more missing observations, and the sample size for the analysis of weight gain was 5383. 746 cohort members reported a high blood pressure diagnosis, and 256 reported developing diabetes since their last interview. Weight change between 2000 and 2012 was severely leptokurtic (most individuals changed weight by roughly the same amount), right skewed, and with a few extreme outliers at both tails of the distribution. Commonly used arithmetic transformations did not yield a normally distributed variable. Recent evidence using the Nurses’ Health cohort study clearly showed that changes in body weight greater than 5 kg in middle-age were associated with negative health outcomes in both men and women: below this cut-off there was not a significant increase in cardiovascular risk [26]. Hence, a binary dependent variable was created based on this cut-off, and the original weight change variable was not used for regression analysis.

Cohort members’ reported reasons for job loss are summarized in Table 1, and descriptive statistics for all of the study variables are reported in Table 2. Table 3 shows Pearson or tetrachoric correlation coefficients between the independent variables. Experiencing job losses was statistically significantly associated with other predictors: women experienced fewer job losses than men, and those with a university degree and more savings had fewer job losses. While many of the predictors were significantly associated with each other, no correlation appears to be substantial enough to result in multicollinearity in the regression models.

**Table 2.** Descriptive statistics for the study variables

Continuous and multiple category variables	Observed minimum	Observed maximum	Mean	Std. deviation	
Weight change between 2000 and 2012 in kg	-104.00	112.70	5.64	9.64	
Number of job losses since 2000	0	8	.19	.50	
Savings reported in 2012 divided by 10 000	0	100	2.42	8.60	
Debt reported in 2012 divided by 10 000	0	50	.48	1.40	
Number of cigarettes a day usually smoked	0	99	2.88	6.61	
Frequency of alcohol consumption (1 = never, 5 = 4+ times per week)	1	5	3.23	1.22	
Number of days in a typical week does 30 min or more of exercise	0	7	2.58	2.35	
Fruit and vegetable consumption (0 = almost never to 20 = fruit, salad, cooked veg, each > once per day)	0	18	10.8	3.33	

Binary variables	Number no	% No	Number Yes		% Yes

Developed diabetes since 2000	9556	97.4	256		2.6
Developed high blood pressure since 2000	9056	92.4	746		7.6
Gained at least 5 kg since 2000	3775	49.9	3787		50.1
Sex of cohort member (1 = male, 2 = female)	4724	48 M	5117		52 F
Has university degree	8225	86.6	1606		13.4

**Table 3.** Pearson or tetrachoric correlations between independent variables (*P*-value in parentheses)

	N. job losses since 2000	Sex (1 = M, 2 = F)	Savings in 2012/ 10 000	Debt in 2012/ 10 000	Uni degree (0 = No, 1 = Yes)	Freq. of having an alcoholic drink	Number of cigarettes a day	N. days 30 min exercise	Freq of eating fruit and vegetables
N. job losses since 2000	1	−.054 (.000)	−.024 (.033)	−.003 (.798)	−.026 (.009)	.000 (.990)	.045 (.000)	−.003 (.761)	−.030 (.006)
Sex (1 = M, 2 = F)	−.054 (.000)	1	−.061 (.000)	−.045 (.000)	−.008 (.447)	−.170 (.000)	−.066 (.000)	−.099 (.000)	.183 (.000)
Savings in 2012/10 000	−.024 (.033)	−.061 (.000)	1	.028 (.014)	.142 (.000)	.081 (.000)	−.057 (.000)	.010 (.392)	.064 (.000)
Debt in 2012/10 000	−.003 (.798)	−.045 (.000)	.028 (.014)	1	.016 (.128)	.018 (.102)	−.027 (.010)	−.021 (.049)	−.010 (.349)
Uni degree (0 = No, 1 = Yes)	−.026 (.009)	−.008 (.447)	.142 (.000)	.016 (.128)	1	.123 (.000)	−.145 (.000)	−.029 (.004)	.184 (.000)
Freq. of having an alcoholic drink	.000 (.990)	−.170 (.000)	.081 (.000)	.018 (.102)	.123 (.000)	1	−.025 (.018)	.043 (.000)	.044 (.000)
Number of cigarettes a day	.045 (.000)	−.066 (.000)	−.057 (.000)	−.027 (.010)	−.145 (.000)	−.025 (.018)	1	−.012 (.234)	−.195 (.000)
N. days does 30 min exercise	−.003 (.761)	−.099 (.000)	.010 (.392)	−.021 (.049)	−.029 (.004)	.043 (.000)	−.012 (.234)	1	.066 (.000)
Freq of eating fruit and vegetables	−.030 (.006)	.183 (.000)	.064 (.000)	−.010 (.349)	.184 (.000)	.044 (.000)	−.195 (.000)	.066 (.000)	1

### Regression results

Tables 4–6 contain summaries of the logistic regression models predicting weight gain of at least 5 kg, diagnosis of high blood pressure since 2000, and diagnosis of diabetes since 2000. The number of job losses experienced by cohort members was significantly associated with odds of a high blood pressure or a diabetes diagnosis. The odds of developing high blood pressure or diabetes increased by 1.28 times and 1.39 times respectively for each reported job loss (see Tables 5 and 6). Other variables in the regression models were significant predictors of all three outcomes: for every 10 000 pounds of reported debt the odds of gaining 5 kg or more, having high blood pressure and developing diabetes increased by 1.09, 1.05 and 1.07 times respectively. In other words, every additional 100 000 pounds of debt approximately doubled the odds of gaining 5 kg or more. Women had a lower odds of all three outcomes, and those who reported doing at least 30 min exercise a day had significantly reduced odds of weight gain and high blood pressure. Having a university degree, and reported fruit and vegetable consumption were associated with lower odds of weight gain and of high blood pressure. The two other lifestyles variables, alcohol consumption and smoking were less successful as predictors: smoking was not a significant predictor in any model, and number of days per week the cohort member reported drinking alcohol was negatively associated with weight gain and diabetes. While this suggests protective effects of alcohol consumption, it should be noted that the number of alcoholic drinks consumed per day was not included in the models.

**Table 4.** Summary of results of logistic regression model predicting weight gain of 5 kg or more between 2000 and 2012

Dependent variable is weight gain of 5 kg or greater	*B*	SE	Odds ratio	95% CI for odds ratio
Number of job losses since 2000	.11	.06	1.11	.99–1.25
Sex of cohort member (1 = M, 2 = F)	-.13	.06	.88	.79–.99
Savings reported in 2012 divided by 10 000	-.01	.01	.99	.99–1.01
Debt reported in 2012 divided by 10 000	.09	.03	1.09	1.04–1.15
Has university degree (0 = No, 1 = Yes)	-.30	.07	.74	.64–.85
Frequency of having an alcoholic drink	-.07	.02	.94	.89–.98
Number of cigarettes a day usually smoked	-.01	.01	.99	.99–1.01
Number of days in a typical week does 30 min or more of exercise	-.09	.01	.92	.90–.94
Frequency of eating fruit and vegetables	-.03	.01	.97	.96–.99
Constant	.81	.13	2.25

**Table 5.** Summary of results of logistic regression model predicting developing high blood pressure between 2000 and 2012

Dependent variable is diagnosis of high blood pressure since 2000	*B*	SE	Odds ratio	95% CI for odds ratio
Number of job losses since 2000	.25	.09	1.28	1.07–1.53
Sex of cohort member (1 = M, 2 = F)	-.26	.11	.77	.63–.95
Savings reported in 2012 divided by 10 000	-.01	.01	.99	.98–1.01
Debt reported in 2012 divided by 10 000	.05	.02	1.05	1.01–1.10
Has university degree (0 = No, 1 = Yes)	-.38	.15	.68	.51–.91
Frequency of having an alcoholic drink	-.04	.04	.96	.88–1.04
Number of cigarettes a day usually smoked	-.01	.01	.85	.98–1.02
Number of days in a typical week does 30 min or more of exercise	-.05	.02	.95	.91–.99
Frequency of eating fruit and vegetables	-.03	.02	.97	.94–.99
Constant	-1.78	.23	.17

**Table 6.** Summary of results of logistic regression model predicting developing diabetes between 2000 and 2012

Dependent variable is diagnosis of diabetes since 2000	*B*	SE	Odds ratio	95% CI for odds ratio
Number of job losses since 2000	.33	.13	1.39	1.08–1.80
Sex of cohort member (1 = M, 2 = F)	-.47	.17	.63	.45–.87
Savings reported in 2012 divided by 10 000	.01	.01	1.01	.99–1.03
Debt reported in 2012 divided by 10 000	.06	.03	1.07	1.01–1.12
Has university degree (0 = No, 1 = Yes)	-.42	.25	.66	.41–1.06
Frequency of having an alcoholic drink	-.39	.07	.67	.59–.77
Number of cigarettes a day usually smoked	-.01	.01	.99	.97–1.02
Number of days in a typical week does 30 min or more of exercise	-.04	.04	.96	.90–1.03
Frequency of eating fruit and vegetables	-.04	.03	.96	.92–1.01
Constant	-1.75	.34	.17	

### Additional analyses

The sensitivity analyses suggested that experiencing at least two job losses was associated with the highest odds of a high blood pressure or a diabetes diagnosis between ages 30 and 42 (see Fig. 1). Too few cohort members experienced at least three job losses, and these sensitivity analyses resulted in wide 95% confidence intervals for this category.

Results of additional analysis of whether the categories of job loss included in the job loss variable have different effects are summarized in Fig. 2. Of particular interest is whether losing a job because a short-term contract ended can be viewed as ‘expected’ stochasticity with lesser health consequences. The results did not support this: the odds ratios were similar to those from analyses of health effects of redundancy. A second category of job loss which may have smaller health consequences is leaving employment after having work hours reduced, or being an independent contractor who is no longer being offered or finding enough work to make a living. Unfortunately the sample size of this type of job loss was small (*n* = 137), but like the short-term contract results, they do not strongly suggest different health outcomes for this category compared with the other two.

## CONCLUSIONS AND IMPLICATIONS

This analysis of participants in the 1970 British Birth Cohort Study demonstrated that employment instability measured as the number of job losses experienced, was associated with increased odds of two risk factors for morbidity and mortality in early middle age: high blood pressure and diabetes. The evidence for a relationship between job losses experienced and weight gain was less robust, although having experienced at least one job loss (as a binary predictor) was associated with gaining at least 5 kg (see Fig. 1). Savings and debt were included as covariates largely because savings and debt may be expected to modify the stress and health risks associated with forced redundancy. Debt in particular was a predictor of weight gain, and increased odds of a high blood pressure or diabetes diagnosis between ages 30 and 42.

Other studies have found similar effects of employment stability on health and known health-risk factors [19–22], but almost none has used longitudinal data on employment histories instead of perceived employment insecurity or the threat of job loss. While belief that one’s job is insecure may have similar relationships with stress and health outcomes, the present study directly suggests that short-term tenure in employment has negative health impacts. The results are consistent with predictions and findings in evolutionary biology of metabolic changes promoting energy storage when resources become unstable in an environment [4].

While the 1970 British Birth Cohort Study has strengths in long-term measurement of economic activity, a weakness with the health data is that blood pressure and diabetes data are binary, self-reported, and have no date or timing of diagnosis other than occurring in the period since last interview. Data using medical records and measured blood pressure and glycated haemoglobin or other diabetes markers would be preferable. A second potential weakness is that loss to follow-up over the decades has largely occurred because participants have moved address and contact has been lost [24]. This means that it is possible that those with the least employment stability have been more likely to be lost to follow-up, and that the estimates of associations between job losses and health reported here underestimate the true effects. Third, changes in weight, blood pressure and blood sugar regulation are likely to have multiple causes, and it is not possible to rule out the observed effects being driven by a third, unobserved, variable or by residual confounding from influential health-related factors such as social class.

For clinicians, this study is another small piece contributing to a large body of evidence that patient histories contain important information about what to look for when making diagnostic and patient care decisions. It is also another small piece of evidence contributing to a large body of evidence that economic policy is health policy.

## DATA SHARING STATEMENT

The data used in this study are available free of charge via the UK Data Service. To create some of the variables used in this research it is necessary to merge data files from different sweeps, and to convert multilevel data to one line of data per participant.

## FUNDING

No external funding was sought or obtained for any of the work involved in preparing the manuscript.

**Conflict of interest:** None declared.
